# The FAK inhibitor BI 853520 inhibits spheroid formation and orthotopic tumor growth in malignant pleural mesothelioma

**DOI:** 10.1007/s00109-018-1725-7

**Published:** 2018-12-11

**Authors:** Viktoria Laszlo, Zsuzsanna Valko, Judit Ozsvar, Ildiko Kovacs, Tamas Garay, Mir Alireza Hoda, Thomas Klikovits, Paul Stockhammer, Clemens Aigner, Marion Gröger, Walter Klepetko, Walter Berger, Michael Grusch, Jozsef Tovari, Irene C. Waizenegger, Balazs Dome, Balazs Hegedus

**Affiliations:** 1 Division of Thoracic Surgery, Department of Surgery, Comprehensive Cancer Center, Medical University of Vienna, Waehringer Guertel 18-20, A-1090 Vienna, Austria; 2 Department of Biomedical Imaging and Image-guided Therapy, Division of Molecular and Gender Imaging, Medical University of Vienna, Vienna, Austria; 3 Department of Tumor Biology, National Korányi Institute of Pulmonology, Budapest, Hungary; 4 2nd Department of Pathology, Semmelweis University, Budapest, Hungary; 5 Department of Thoracic Surgery, Ruhrlandklinik, University Clinic Essen, University Duisburg-Essen, Tüschener Weg 40, 45239 Essen, Germany; 6 Core Facility Imaging, Medical University of Vienna, Vienna, Austria; 7 Institute of Cancer Research and Comprehensive Cancer Center, Department of Medicine I, Medical University of Vienna, Vienna, Austria; 8 Department of Experimental Pharmacology, National Institute of Oncology, Budapest, Hungary; 9 KINETO Lab Ltd, Budapest, Hungary; 10 Boehringer Ingelheim RCV GmbH & Co KG, Vienna, Austria; 11 Department of Thoracic Surgery, National Institute of Oncology-Semmelweis University, Budapest, Hungary

**Keywords:** Mesothelioma, Focal adhesion kinase, Tyrosine kinase inhibitor, Angiogenesis, Spheroid formation, Orthotopic xenograft

## Abstract

**Abstract:**

No tyrosine kinase inhibitors are approved for malignant pleural mesothelioma (MPM). Preclinical studies identified focal adhesion kinase (FAK) as a target in MPM. Accordingly, we assessed the novel, highly selective FAK inhibitor (BI 853520) in 2D and 3D cultures and in vivo. IC_50_ values were measured by adherent cell viability assay. Cell migration and 3D growth were quantified by video microscopy and spheroid formation, respectively. Phosphorylation of FAK, Akt, S6, and Erk was measured by immunoblot. The mRNA expression of the putative tumor stem cell markers SOX2, Nanog, CD44, ALDH1, c-myc, and Oct4 was analyzed by qPCR. Cell proliferation, apoptosis, and tumor tissue microvessel density (MVD) were investigated in orthotopic MPM xenografts. In all 12 MPM cell lines, IC_50_ exceeded 5 μM and loss of NF2 did not correlate with sensitivity. No synergism was found with cisplatin in adherent cells. BI 853520 decreased migration in 3 out of 4 cell lines. FAK phosphorylation was reduced upon treatment but activation of Erk, Akt, or S6 remained unaffected. Nevertheless, BI 853520 inhibited spheroid growth and significantly reduced tumor weight, cell proliferation, and MVD in vivo. BI 853520 has limited effect in adherent cultures but demonstrates potent activity in spheroids and in orthotopic tumors in vivo. Based on our findings, further studies are warranted to explore the clinical utility of BI 853520 in human MPM.

**Key messages:**

- Response to FAK inhibition in MPM is independent of NF2 expression or histotype.
- FAK inhibition strongly interfered with MPM spheroid formation.
- BI 853520 has been shown to exert anti-tumor effect in MPM.

**Electronic supplementary material:**

The online version of this article (10.1007/s00109-018-1725-7) contains supplementary material, which is available to authorized users.

## Introduction

Malignant pleural mesothelioma (MPM) is a highly aggressive malignancy with a strong link to prior asbestos exposure. Invasive growth, locoregional spread, and frequent local recurrence are considered to be responsible for its dismal overall survival (OS), which ranges from 9 to 14 months [1]. Despite the modest OS benefit achieved by adding bevacizumab to standard cisplatin plus pemetrexed chemotherapy [2], no other molecularly targeted drugs could so far enter the clinical practice for this fatal malignancy. Focal adhesion kinase (FAK), also known as protein tyrosine kinase 2 (PTK2), is a ubiquitously expressed 125-kDa non-receptor tyrosine kinase which consists of three distinct domains: a central catalytic domain, a C-terminal FAT domain, and an N-terminal FERM domain [3, 4]. FAK is located in the cytosol, where it is particularly prominent in focal adhesions that interact with different extracellular matrix components [5]. Accordingly, during homeostatic conditions, FAK activation mainly relies on signals from integrins and growth factor receptors, leading to autophosphorylation of the Y397 site in the N-terminal domain [3, 4, 6–8]. FAK overexpression has been preferentially linked to a more aggressive tumor behavior, particularly by promoting tumor cell proliferation, survival, motility, invasion, stem cell renewal, angiogenesis, and metastasis [4, 6, 7, 9–11]. In this context, there is emerging evidence for a functional role of FAK gene amplification and protein overexpression during tumor progression in different tumor types including lung, breast, colorectal, thyroid, kidney, and pancreatic cancers as well as astrocytoma and osteosarcoma [10, 12–14]. Of note, recent studies demonstrated that FAK activation is an important regulator of the immunosuppressive tumor microenvironment and thus promotes immune evasion in murine models of squamous cell carcinoma and pancreatic cancer [15, 16].

Recognition of the crucial role of FAK in the aforementioned tumor promoting functions prompted the development of FAK small molecule inhibitors and their application for cancer therapy [3, 6, 9, 14]. Early preclinical data provided a clear scientific rationale for the anti-tumorigenic effects mediated by FAK inhibitors, though concerns were raised about their potential toxicity via interacting with ATP binding sites on other tyrosine kinases [3]. This led to the development of novel small molecules with high selectivity for FAK which can prevent the autophosphorylation [3, 11]. These ATP-competitive small molecule inhibitors are effective in preclinical models representing a variety of malignancies including MPM [17–19].

Merlin, a frequently inactivated tumor suppressor protein in MPM, is encoded by the neurofibromatosis type 2 (NF2) gene [20]. Merlin-deficient MPMs exhibit increased FAK expression and tumor cell invasion [21, 22]. In a recent clinical phase I study, MPM patients progressing rapidly on prior treatments showed prolonged stable disease when treated with GSK2256098, a specific FAK inhibitor. Interestingly, in this study, patients with merlin-deficient tumors had a significantly better progression-free survival (PFS) than those with merlin-positive tumors [23]. Another study showed similarly better PFS in an advanced MPM patient with unknown merlin status treated with VS-6063, also known as defactinib [24]. These clinical studies are in line with in vitro data, where the FAK inhibitor VS-4718 prominently reduced proliferation and triggered apoptosis in merlin-deficient tumor cells [19]. Notably, Kato et al. reported that E-cadherin expression is a predictive biomarker for VS-4718 FAK in merlin-negative MPM patients [25]. However, a recent phase 2, double-blinded, placebo-controlled study (COMMAND) investigating another FAK inhibitor (VS-6063) in MPM patients randomized by merlin status of the tumor had to be prematurely stopped due to futility (NCT01870609).

Based on these controversial studies, further preclinical investigations are necessary to better understand the interplay between FAK and E-cadherin functions particularly in MPM and, furthermore, to identify novel markers that help to stratify patients for FAK inhibitor susceptibility.

BI 853520 is a novel highly specific FAK inhibitor that has previously demonstrated a good oral bioavailability in mice and led to tumor growth inhibition in a panel of 21 different human carcinomas and in human fibrosarcoma at doses as low as 6 mg/kg [26, 27]. This is the first study investigating the anti-tumor potential of BI 853520 in different in vitro and in vivo models of human MPM.

## Materials and methods

### Cell culture

SPC111, SPC212, and M38K cell lines were initially established from human biphasic MPMs, kindly provided by Prof. R. Stahel (SPC111 and SPC212, University of Zurich, Zurich, Switzerland) and Prof. V.L. Kinnula (M38K, University of Helsinki, Helsinki, Finland). The P31 epithelioid MPM cell line was kindly provided by Prof. K. Grankvist (University of Umea, Umea, Sweden). The non-malignant mesothelial cell line Met5a (ATCC CRL-9444) was purchased from the American Type Culture Collection. Meso and VMC primary cell lines were established at the Medical University of Vienna (ethics approval number: “THORAXBANK” 904/2009). Histological subtype distribution among these cell lines are listed in Table 1. Cells were cultured in RPMI-1640 or DMEM plus 10% fetal calf serum (FCS) (Sigma Chemical Co., St. Louis, MO), 100 U/ml penicillin, and 10 mg/ml streptomycin (Sigma Chemical Co., St. Louis, MO). Cell lines were kept at 37 °C in a humidified incubator with 5% CO_2_.

**Table 1:** Histological subtype, merlin status, and IC_50_ values for each cell line

Cell line	Histological subtype	Merlin status	IC_50_ of BI 853520 (μM)
Met5a	Immortalized mesothelial cell	−	9
Meso49	Epithelioid MPM	−	5.5
Meso53	Biphasic MPM	+	5.5
SPC111	Biphasic MPM	−	5.6
P31	Epithelioid MPM	−	5.9
VMC20	Epithelioid MPM	−	5.9
VMC23	Epithelioid MPM	−	5.8
M38K	Biphasic MPM	+	6.6
VMC40	Biphasic MPM	−	7.5
Meso62	Sarcomatoid MPM	−	8.8
SPC212	Biphasic MPM	+	10.2
VMC6	Epithelioid MPM	−	11.5
VMC12	Epithelioid MPM	+	11.3

### Chemosensitivity assays

In order to assess the short-term effect of BI 853520 (Boehringer Ingelheim) on cell viability, total protein amount–based sulforhodamine B (SRB) assays were performed in 12 MPM cell lines and in the immortalized mesothelial line Met5a. Additionally, BI 853520 and cisplatin (Teva Hungary) combination treatments were done in one merlin-positive (SPC212) and one merlin-negative cell line (P31). SRB assays were executed as published previously [28]. Briefly, cells were seeded in 96-well plates 24 h before drug exposure to be afterwards treated with different concentrations of cisplatin and BI 853520 for 72 h. Regarding combination treatments, we calculated combination indices (CI) according to Chou and Talalay [29] with the CalcuSyn software (Biosoft, Ferguson, MO). CI values < 0.9, from 0.9 to 1.1, or > 1.1 indicated synergism, additive effects, or antagonism between BI 853520 and cisplatin, respectively.

### Analysis of in vitro cell migration by time-lapse video microscopy

2D video microscopy measurements were performed and analyzed as previously described [30]. Briefly, cells were seeded in 24-well plates (Corning Incorporated, Corning, NY) and cultured in DMEM medium supplemented with 10% FCS. Medium was changed to CO_2_-independent medium (Invitrogen, Carlsbad, CA) with 10% FCS and 4 mM glutamine, and cells were kept in a custom-designed incubator built around an inverted phase-contrast microscope (World Precision Instruments, Sarasota, FL). Every 5 min, images from three distinct neighboring microscopic fields were taken for at least 48 h. After 24 h of observation, BI 853520 was added and cells were finally observed for another 72 h. Individual cells were tracked for the first 24 h after treatment with a cell tracking program, their position parameters were extracted, and migrated distance was calculated as previously described [31].

### Tumor cell spheroid assay

In order to obtain MPM cell line–derived spheroid cultures, 5 × 10^3^ MPM cells (SPC212 or P31) were seeded in triplicate in DMEM/Ham’s F-12 medium (Biochrom) in ultra-low attachment 24-well plates (Thermo Scientific). Medium was supplemented with 20 ng/ml basic fibroblast growth factor (FGF) (PeproTech), 20 ng/ml epidermal growth factor (Sigma), and 2% B27 supplement (Gibco). BI 853520 was added at 0.1 μM and 1 μM. Ninety-six hours following plating, spheroids in all wells were photographed. Spheres with a diameter above 100 μm were counted. Diameters were measured by using ImageJ software.

### Western blot analysis

For merlin and E-cadherin expression analysis, subconfluent cultures of all 12 cell lines were processed. To determine the impact of FAK inhibition on cell signaling, SPC111, P31, SPC212, or M38K cells were seeded into 6-well plates and—after 24 h of recovery—treated with BI 853520 or solvent. Subsequently, cells were extracted in RIPA buffer (Fisher Scientific, Waltham, MA, Cat. No.: 3168890) containing protease inhibitor cocktail (Fisher Scientific, Cat. No.: 78430) to later separate proteins by SDS-PAGE. Proteins were blotted onto nitrocellulose membranes and immunostained with the following primary antibodies from cell signaling: merlin (#6995), E-cadherin (#3195), Erk (#9102), p-Erk (#9101), Akt (#9272), p-Akt (#4058), S6 (#2215), p-S6 (#2217), FAK (#3285), p-FAK (Tyr-397, #8556), and β-tubulin (#2128). For antibodies, a dilution of 1:1000 was applied, followed by horseradish peroxidase–coupled secondary antibody (Thermo) incubation and development by ECL Reagent (GE Healthcare, GE Healthcare, Dassel, Germany) with a developer machine (Curix60 AGFA Type 9462/106).

### Analysis of tumor stem cell marker expression

For tumor spheroid formation, MPM cells were seeded as described above and treated with 0.1 μM and 0.5 μM BI 853520 for 4 days. Total RNA was isolated with TRIzol and reverse transcribed with MMLV reverse transcriptase (Thermo Fisher Scientific) and random hexamer primers. The resulting cDNAs were analyzed using TaqMan gene expression assays on the Applied Biosystems 7500 Fast Real-Time PCR System (Assay IDs: ALDH1A1 Hs00946916; CD44 Hs01075854; GAPDH Hs02786624; Myc Hs01570247; NANOG Hs02387400; Oct4 Hs04260367; SOX2 Hs01053049; Thermo Fisher Scientific). Gene expression was calculated by the ΔΔC(T) method using GAPDH as reference gene.

### Orthotopic in vivo MPM xenograft model

In order to develop orthotopically growing MPM tumors, two million P31 cells were inoculated into the chest cavity of 8-week-old female SCID mice from our colony. Animals were randomly stratified into treatment and control groups once tumor nodules had a macroscopically visible size (based on our preliminary experiments 28 days following tumor implantation). Regarding treatment, mice received solvent or 20 mg/kg BI 853520 (dissolved in methylcellulose) orally five times a week on consecutive days (one cycle with 5 days on and 2 days off treatment). Animals were weighed three times a week and the experiment was ended on the 20th day of treatment. Finally, MPM tumor nodules were harvested, weighed, and fixed in formalin and embedded in paraffin (FFPE) or stored as frozen tissue. All mentioned animal experiments were performed according to the ARRIVE guidelines [32] and to the animal welfare regulations of the host institutes (permission number: PEI/001/2574-6/2015).

### Evaluation of apoptosis and tumor vascularization in vivo

Serial frozen sections were cut from each tumor. For the analysis of apoptosis, terminal deoxynucleotidyl transferase–mediated dUTP nick-end labeling (TUNEL) was accomplished according to the manufacturer’s instructions (Roche Diagnostics). DAPI was used to label nuclei and slides were scanned by TissueFAXS (TissueGnostics GmbH, Vienna, Austria). Apoptosis was quantified as the percentage of TUNEL-positive nuclei among DAPI-labeled ones. To analyze the effect of FAK inhibition on angiogenesis, consecutive frozen sections were stained for microvessels with CD31 (clone SZ31, Dianova). Intratumoral microvessel density analysis was performed by using the StrataQuest software (TissueGnostics, Vienna, Austria).

### Assessment of tumor cell proliferation by Ki67 labeling

Ki67 immunohistochemistry was performed as described earlier [33]. Three-micrometer sections from formalin-fixed and paraffin-embedded (FFPE) tumor nodules were deparaffinized and rehydrated with decreasing alcohol concentrations. Following heat-induced antigen retrieval in citrate buffer (pH 6.0), slides were incubated with the Ki67 antibody (mouse mAb, Dako Cytomation, clone MIB-1, dilution 1:100) for 30 min. UltraVision LP detection system (Lab Vision Corporation, Fremont, CA) was used to detect antibody binding according to the manufacturer’s recommendations. 3-3-Diaminobenzidine (Dako) was applied for color development. Each analyzed slide was counterstained with Mayer’s hematoxylin (Sigma) and images were taken using a bright-field microscope (Axio Imager, Carl Zeiss). Labeling index was determined by using the automated image analysis application ImmunoRatio [34]. At least 2000 tumor cells were counted to obtain the mean percentage of Ki67-positive MPM tumor cells per sample.

### Statistical analyses

In order to compare two groups, Mann-Whitney *U* tests were performed. Kruskal-Wallis and Dunn’s multiple comparison tests were used for more than two groups. *P* values below 0.05 were considered statistically significant. For all statistical analyses, the GraphPad Prism 5.0 software (GraphPad Inc., San Diego, CA) was applied.

### Data accessibility

Data and commercially not available material is available from the corresponding authors upon reasonable request.

## Results

### Response to FAK inhibition in adherent MPM cell models in vitro is independent of merlin expression and histological subtype

**Fig. 1:**
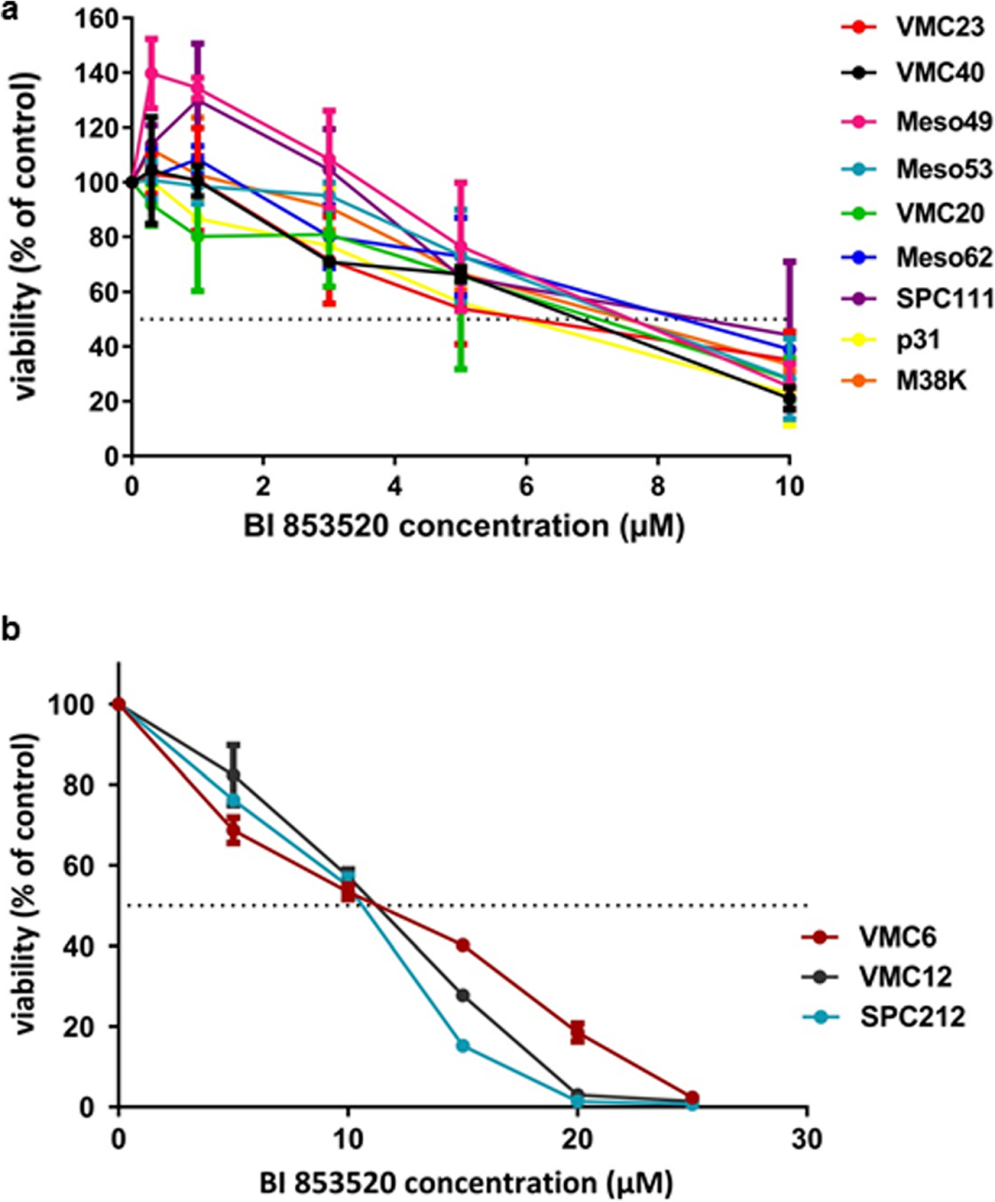
BI 853520 has limited growth inhibitory potential in 2D MPM cell cultures. MPM cells (*n* = 12) were treated with different concentrations of BI 853520 and incubated for 72 h. Dose-response curves (mean ± SEM) from three independent experiments are presented for more sensitive (**a**) and more resistant (**b**) cell lines

In order to investigate whether histological subtype and merlin or E-cadherin expression influence the sensitivity of MPM cells to FAK inhibition in vitro, we determined the IC_50_ values by performing SRB assays of adherently grown cells following 72 h of BI 853520 treatment (Fig. 1 and Table 1). None of the 12 MPM cell lines in our panel expressed E-cadherin (data not shown). All cell lines demonstrated IC_50_ values above 5 μM including three cell lines with very low sensitivity (IC_50_ values above 10 μM, Fig.1b). In our panel of cell lines, sensitivity to BI 853520 showed no association with histological subtype or merlin expression (Supplementary Fig. 1).

### BI 853520 does not synergize with cisplatin in vitro

Since certain FAK inhibitors demonstrated a synergistic effect with cisplatin in other malignant diseases [9], cell viability was also measured when BI 853520 was applied in combination with cisplatin. In these experiments, we used the relatively sensitive and merlin-negative P31 and the more resistant and merlin-expressing SPC212 cells. The CI index calculations demonstrate that at the majority of concentrations, only additive or even antagonistic interactions are achievable (Supplementary Fig. 2).

### FAK inhibition reduces MPM cell motility in a cell type–dependent manner

**Fig. 2:**
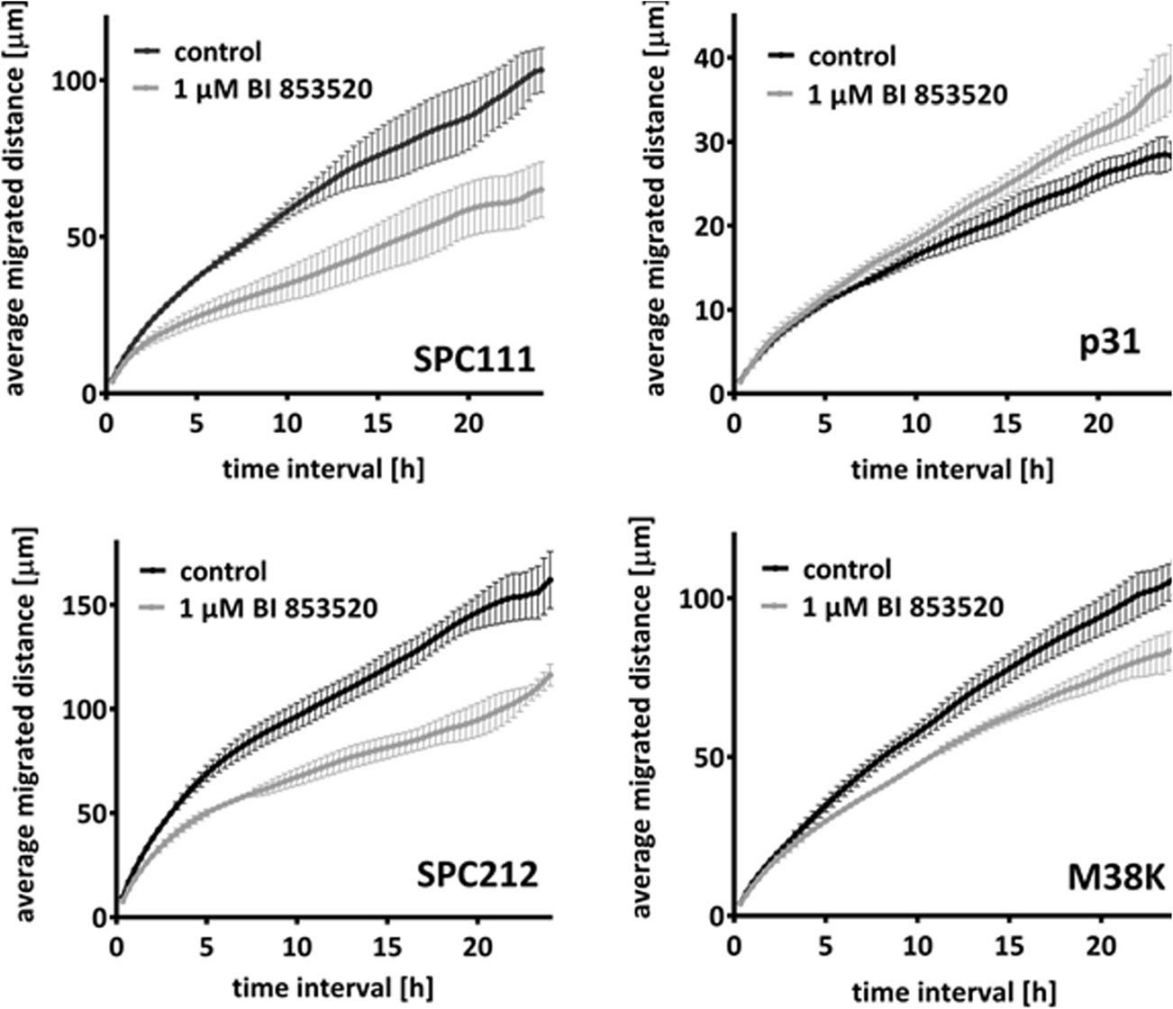
Cell line–dependent migratory response to BI 853520 treatment. SPC111, SPC212, P31, and M38K cell cultures were treated with 1 μM BI 853520 or solvent in CO_2_-independent medium supplemented with 10% FBS. Cell migration was observed by video microscopy for 72 h. Average migrated distance was analyzed in the 48- to 72-h period after treatment. BI 853520 treatment reduced the migratory potential of SPC111, SPC212, and M38K cells. Interestingly, a slight increase in the migration of P31 cells could be seen

Because FAK is a known regulator of actin remodeling and focal adhesion dynamics, we also measured the in vitro migratory activity in the presence of BI 853520. The cell lines SPC111, SPC212, P31, and M38K were selected for the further experiments to represent both epithelioid and biphasic and merlin-expressing as well as merlin-negative subtypes. The average migrated distance of the four different MPM cell lines was measured in the first 24 h following treatment with BI 853520 or solvent (Fig. 2). Of note, BI 853520 showed an anti-migratory effect even at the 1 μM concentration which is way below the IC_50_ values for cell viability in three of the four cell lines. Interestingly, SPC212 cells with very low sensitivity in adherent growth assay demonstrated a strong migration inhibitory response. The cell line with the lowest baseline motility, i.e., P31, showed a slight increase in migration at this concentration.

### BI 853520 efficiently reduces FAK phosphorylation but not the activation of Akt, S6, or Erk

**Fig. 3:**
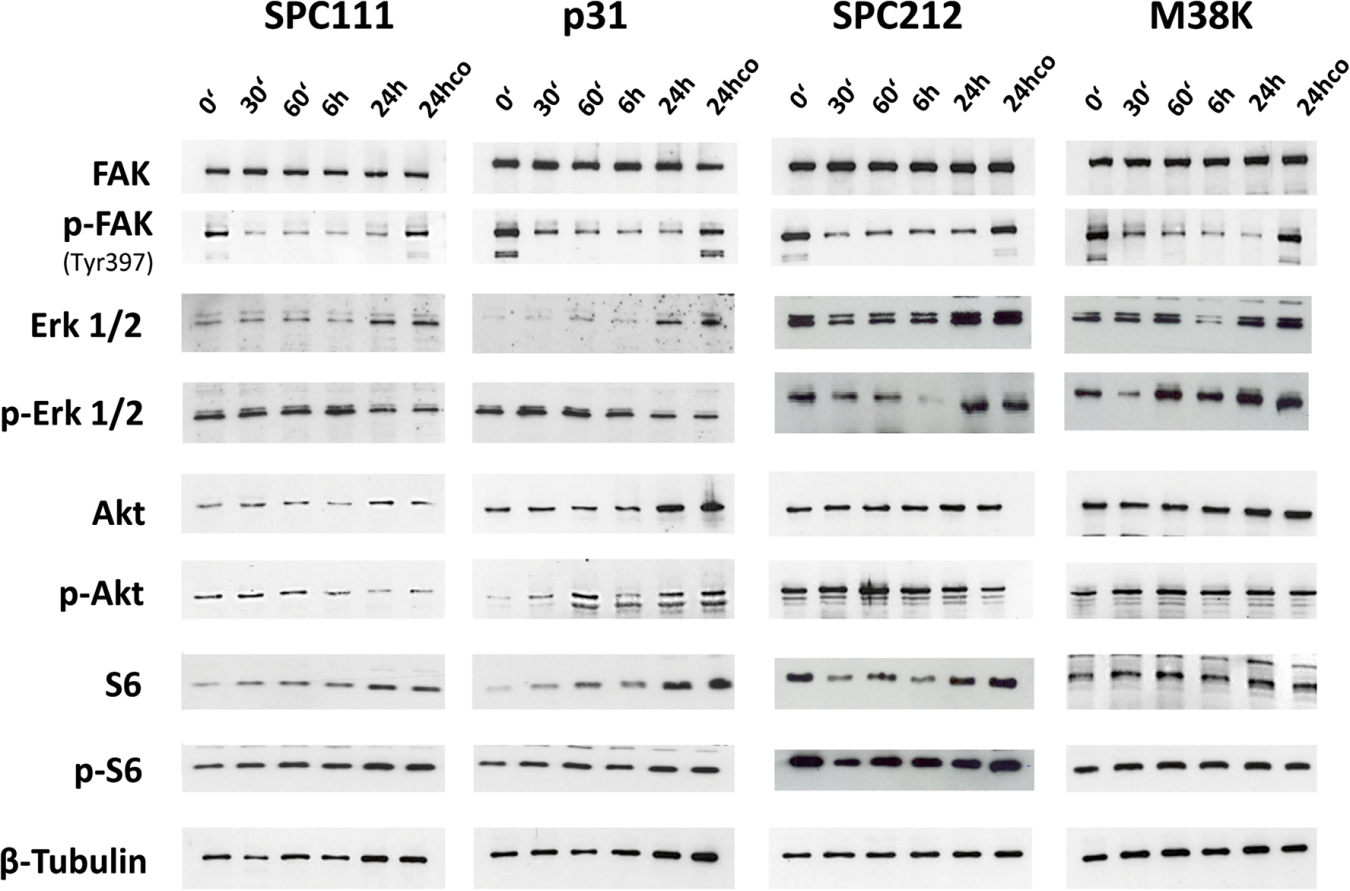
BI 853520 reduces FAK phosphorylation without major impact on Erk, Akt, or S6 activation in vitro. Time-dependent Western blot assays illustrate the impact of 1 μM BI 853520 treatment on Erk1/2, Akt, S6, and FAK phosphorylation in two merlin-expressing (SPC212, M38K) and two merlin-negative MPM cell lines (SPC111, P31). While FAK phosphorylation on Tyr397 was efficiently inhibited in all four cell lines, no downstream inactivation was seen in Erk, Akt, or S6. As loading control β-tubulin was applied

In order to examine if FAK is activated in MPM cells and whether BI 853520 can interfere with FAK phosphorylation, we performed time-course immunoblot analyses in adherent cultures (Fig. 3 and Supplementary Fig. 3). While the FAK inhibitor led to a rapid and sustained decrease (i.e., from 30 min to 24 h) of Tyr397 phosphorylation in the four cell lines studied (SPC111, SPC212, P31, and M38K), we did not observe a sustained and consequent reduction in Akt, S6, or Erk phosphorylation. After 24 h of BI 853520 treatment, the FAK phosphorylation remained inhibited but there was no difference in the cells in downstream activation when compared to the 24-h solvent-treated controls.

### FAK inhibition interferes with MPM cell spheroid formation

**Fig. 4:**
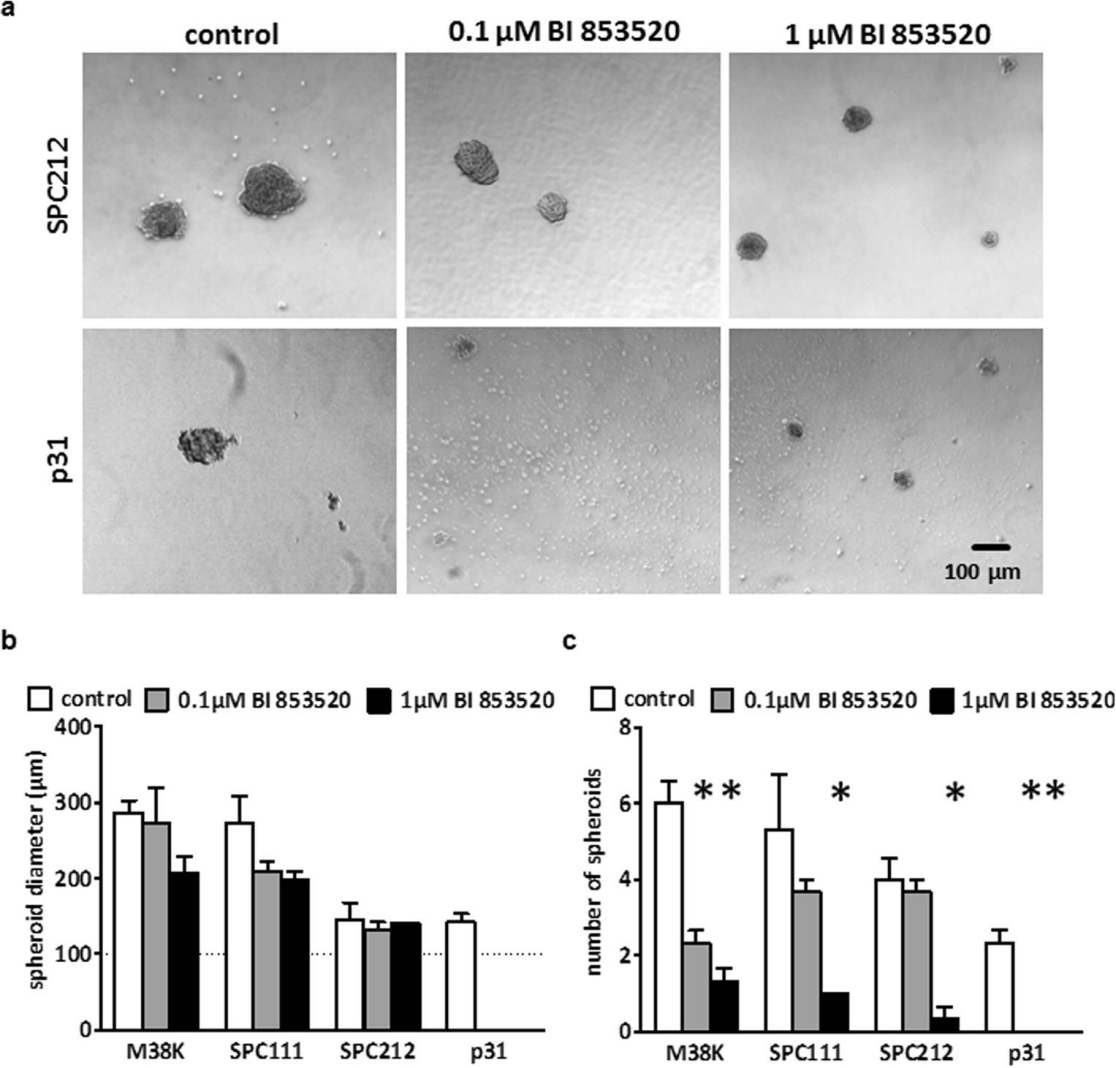
BI 853520 inhibits MPM cell spheroid growth. **a** Representative photomicrographs demonstrating the impact of BI 853520 treatment on spheroid growth following 96 h of drug exposure. **b**, **c** There was no significant change in spheroid diameters, but a significant dose-dependent reduction in spheroid numbers was seen. For all conditions, at least three independent experiments were performed. Bars represent mean ± SEM and asterisks indicate significant (*P* < 0.05) differences when compared to control

Because FAK inhibitors were previously found to have an impact on tumor-initiating cell populations [19], we performed spheroid formation assays by culturing SPC111, SPC212, P31, and M38K cells under serum-free and non-adherent conditions (Fig. 4). While all four cell lines readily formed spheroids after 96 h under control conditions, we observed a cell line–dependent significant reduction in spheroid numbers at concentrations lower than the IC_50_ values pertinent to adherent MPM cell growth. P31 cells were not capable of forming spheroids in the presence of 0.1 or 1 μM BI 853520. Of note, despite the significant reduction in spheroid numbers, the diameter of the spheroids was not affected by BI 853520 in SPC212 cells. Importantly, similar to the adherent cell cultures, 24-h FAK inhibition led to a decrease in the phosphorylation of Tyr397 in all four cell lines; however, no reduction in Akt, S6, or Erk phosphorylation was observed in spheroid cultures (Supplementary Fig. 4). Notably, treatment of MPM spheroids with 0.1 or 0.5 μM BI 853520 did not change the expression of the putative tumor stem cell markers SOX2, Nanog, CD44, ALDH1, c-myc, and Oct4 (Supplementary Fig. 5).

### BI 853520 reduces growth, proliferation, and microvessel density of orthotopically growing human MPM xenografts in mice

**Fig. 5:**
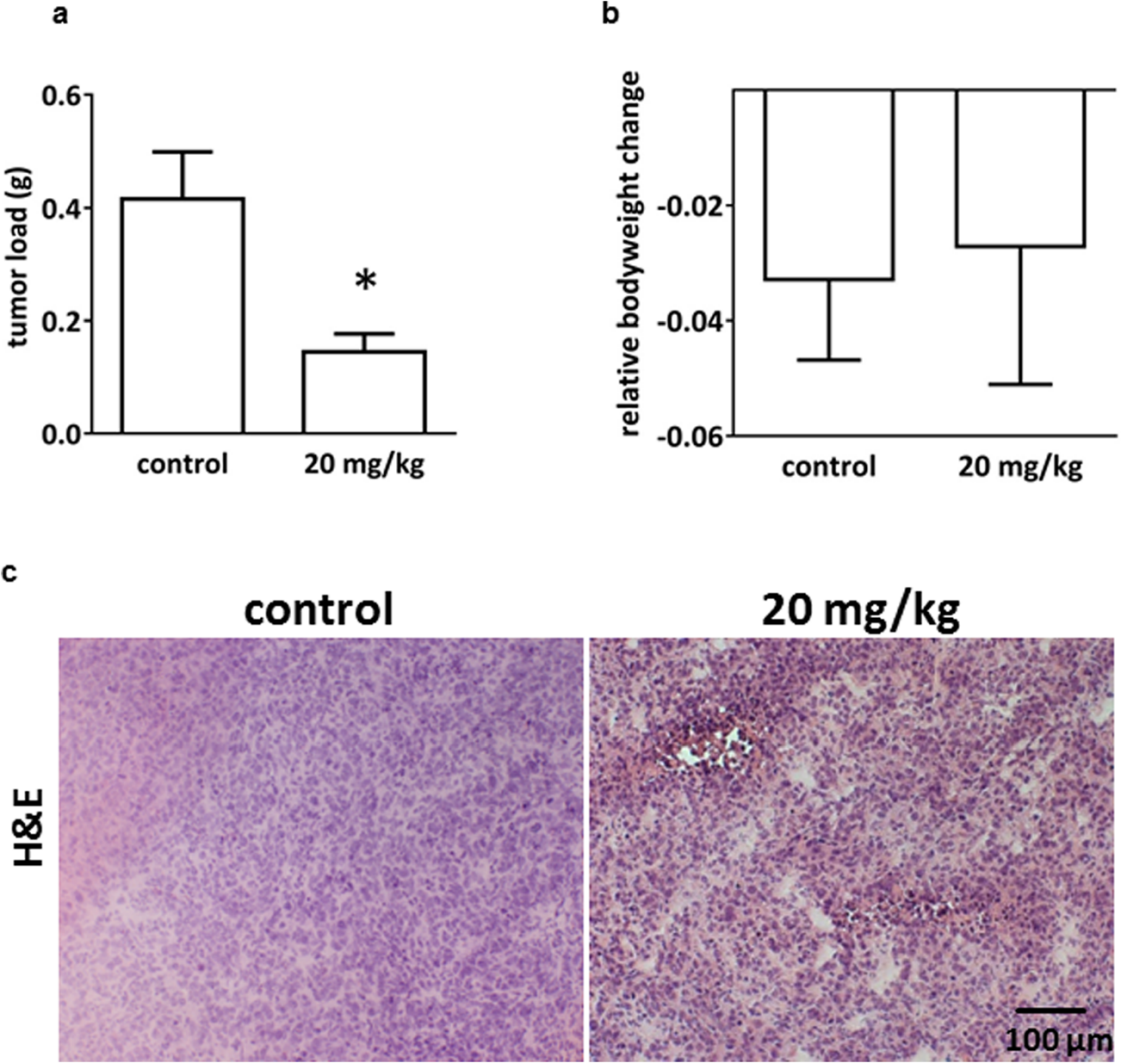
In vivo effect of BI 853520 in orthotopically growing human MPM xenografts. **a** P31 cells were orthotopically injected into SCID mice. Following 28 days of tumor inoculation, 20 mg/kg BI 853520 or solvent was administered *orally* for 3 weeks five times per week. After treatment, mice were sacrificed and their MPM tumor load was quantified. Tumor-bearing mice treated with BI 853520 had significantly lower tumor loads (vs. solvent-treated controls; *P = 0.0183). **b** Relative body weight was decreased by the time of termination, but no significant difference was found between BI 853520- and solvent-treated animals. **c** Microscopy images of representative hematoxylin and eosin (H&E) staining in MPM xenograft tumors of either BI 853520- or solvent-treated mice. Scale bar 100 μm

**Fig. 6:**
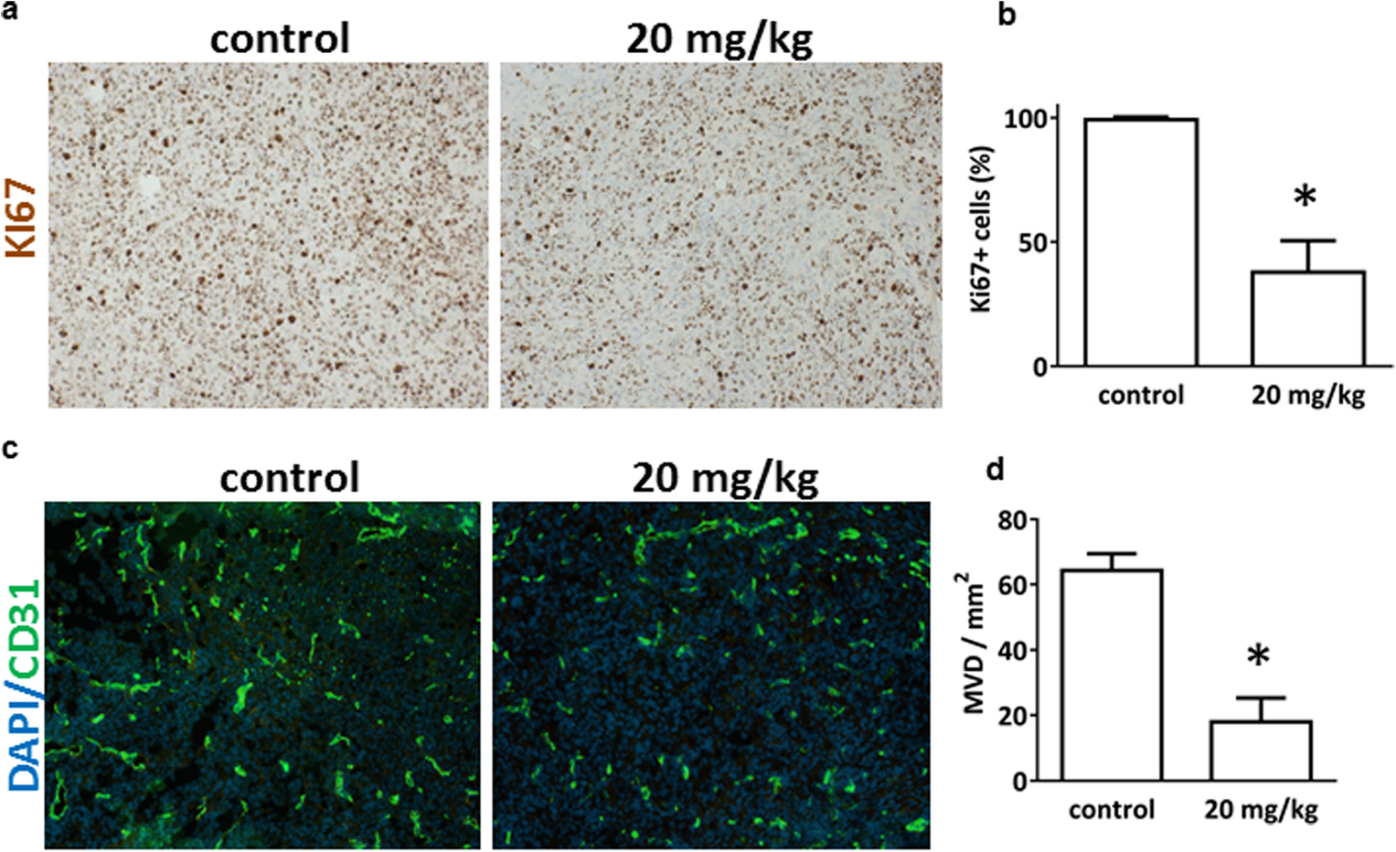
BI 853520 inhibits tumor cell proliferation and angiogenesis in orthotopically growing human MPM in mice. **a** Representative images of Ki67 immunohistochemistry in MPM xenograft tumors of either BI 853520- or solvent-treated tumor-bearing mice. Scale bar 100 μm. **b** Quantification of Ki67-labeling indicates significant inhibitory effect of BI 853520 treatment on MPM cell proliferation (*P* = 0.0012). **c** Impact of BI 853520 treatment on tumor angiogenesis. Representative immunofluorescence images of frozen sections from control and BI 853520-treated tumors labeled with DAPI (blue) and anti-CD31 Ab (green). **d** There was a significant reduction in microvessel densities (MVDs) in the BI 853520-treated (20 mg/kg) group (*P* = 0.0159)

In order to investigate whether the in vitro effects translate to in vivo inhibitory potential, an orthotopic MPM model was established by injecting P31 cells—the cell line with the strongest response to FAK inhibition in spheroid formation—into the thoracic cavity of immunocompromised SCID mice (Fig. 5). There was a significant reduction in the tumor load as quantified by the total tumor weight dissected from the thoracic cavity after 3 weeks of per OS treatment with 20 mg/kg BI 853520 in a schedule of 5 days on and 2 days of (Fig. 5a). Notably, BI 853520 treatment did not influence weight loss in the mice (Fig. 5b). Figure 5c shows representative hematoxylin and eosin (H&E) staining in xenograft tumors of either BI 853520- or solvent-treated mice. Mechanistically, the proliferation of tumor cells in the orthotopic tumors was decreased significantly in the BI 853520-treated animals (Fig. 6a, b); however, we did not observe increased apoptosis (as detected by TUNEL labeling) in these tumors (Supplementary Fig. 6). Importantly, vascularization of human MPM xenograft tumors was significantly reduced by BI 853520 treatment (Fig. 6c, d).

## Discussion

In the current study, we demonstrate the impact of the FAK inhibitor BI 853520 on MPM tumor growth in 2D and 3D in vitro cultures and in an in vivo orthotopic model of human MPM. FAK is a multifunctional non-receptor protein tyrosine kinase, and its role in the progression of a variety of cancer types has now been abundantly demonstrated [35]. Importantly, inhibiting FAK by using small molecule inhibitors—such as BI 853520—that prevent the autophosphorylation seems to be a reliable approach [3, 11].

Relatively high IC_50_ values were measured in our panel of cell lines in adherent cell viability assays. In contrast, BI 853520 treatment could interfere with spheroid formation at much lower concentrations. In support of this, another FAK inhibitor, namely PND-1186, was reported to selectively induce cancer cell apoptosis in a 3D environment [36]. Similarly, the FAK inhibitor VS-6062 demonstrated a greater effect in anchorage-independent growth of ovarian cancer cells when compared to cells in adherent cultures [18]. Hence, the significant 3D growth inhibitory effect of BI 853520 in human MPM cultures is important because there is accumulating evidence in drug development suggesting that 3D tumor cell cultures (vs. 2D monolayers) more accurately model in vivo tumor complexity and thus provide more reliable data for subsequent in vivo and clinical testing [37, 38]. MPM progression is especially relevant in this regard because tumor spheroids are often present in the pleural effusions of MPM patients and contribute to tumor spread within the pleural cavity [39].

Cancer stem cells or tumor-initiating cells are defined as a small subpopulation of tumor cells with the potential to self-sustain and also to maintain tumor growth [19]. Previous studies in different tumor types (including MPM) [19] put forward the hypothesis that FAK inhibitors might preferentially target tumor-initiating cells that are capable of non-adherent growth and are thought to be enriched in tumor spheroids [19, 35]. BI 853520 treatment, however, did not alter tumor stem cell marker (SOX2 [40], ALDH1 [19, 40], NANOG [40], c-myc [40], CD44 [41], Oct4 [40, 41]) expressions of human tumor spheroids formed by different MPM cell lines. Based on our results, BI 853520 thus cannot be considered a selective inhibitor of tumor-initiating cells in human MPM spheroids.

FAK inhibitor sensitivity has been linked to merlin and E-cadherin expression in previous studies [18, 19, 25]. E-cadherin was found to be responsible for FAK inhibitor treatment failure in NF2-deficient MPM cells [25]. This is congruent with an in vitro study in bladder cancer, in which E-cadherin knockdown led to restoration of FAK inhibitor–mediated suppression of cancer cell invasion and migration [42]. Unfortunately, in our panel of cell lines, there were no E-cadherin-expressing MPM cells preventing us to study its impact on treatment responsiveness. With regard to NF2 expression, we found no significant difference in the adherent growth inhibitory potential of BI 853520; however, only four NF2-expressing cell lines were present in our panel.

The majority of MPM cell lines have considerable migratory activity, and integrin-mediated cell adhesion is reorganized during malignant progression [28, 30, 43]. In line with these data, in the current study, FAK inhibition led to a decrease in MPM migration in three out of four tested MPM cell lines. Since direct pharmacological targeting of integrins remains challenging, interfering with FAK as a potent downstream kinase might represent a viable approach to indirectly inhibit certain integrin functions.

Despite the relatively high BI 853520 IC_50_ values observed in our adherent cell growth assays, BI 853520 alone resulted in significantly decreased tumor load and tumor cell proliferation in an orthotopic model of human MPM. This is in line with previous studies using other FAK inhibitors in mouse models of human MPM [19]. Importantly, we could demonstrate that BI 853520 inhibits in vivo angiogenesis. This finding further supports the notion that FAK regulates endothelial cell migration and promotes angiogenesis and thus can serve as an additional target for anti-angiogenic cancer therapy [44]. The promising results from trials using first-line bevacizumab, cisplatin, and pemetrexed combinations in MPM patients underline the importance of anti-angiogenic therapies in this particular malignancy [45]. Importantly, FAK as a downstream element integrates proangiogenic signals received both via VEGFRs and integrin receptors, and as such, it might further increase the efficacy of VEGFR-directed therapeutic modalities [9, 46]. For example, in vivo studies in pancreatic neuroendocrine tumors indicated reduced incidence and number and size of liver metastases following the combination of another FAK inhibitor (OXA-11) with VEGFR2 blockade [9]. Additionally, a recent study on ovarian cancer mouse models showed that combining anti-angiogenic agents with FAK inhibitors clearly reduces tumor growth and, moreover, that FAK inhibitor treatment following anti-angiogenic therapy withdrawal inhibits tumor rebound [46].

Previous studies indicated that FAK inhibitors might enhance the effect of various chemotherapeutic agents such as paclitaxel and cisplatin in ovarian cancer and pancreatic neuroendocrine tumors, respectively [9, 47]. In the current study, two cell lines were tested in combination with cisplatin, but no synergisms were detected in adherent growth assays. Nevertheless, the combination of BI 853520 with other cytotoxic or targeted treatment modalities directly reducing MPM cell survival might prove to be efficient in future studies. For instance, we have recently reported that MPM cells co-express various FGFs and FGF receptors and, moreover, that inhibition of FGF receptor activity reduces MPM cell growth and migration and synergizes with chemo- and radiotherapy in vivo [48]. Because novel multitarget inhibitors have already been developed that can target certain tyrosine kinases and FAK simultaneously (e.g., PHM16, a novel dual FAK/FGFR2 inhibitor with potent anti-tumor and anti-angiogenic activities [49]), the simultaneous inhibition of FAK and FGFRs is a promising novel approach that should be investigated in human MPM models as well.

The recent phase 3 MAPS (Mesothelioma Avastin Cisplatin Pemetrexed Study) trial demonstrated increased OS in unresectable MPM from adding bevacizumab to standard-of-care chemotherapy [2], indicating that targeting the tumor vasculature might be an effective anti-MPM strategy. Despite this development, the prognosis of MPM remains dismal, and no additional molecularly targeted therapeutic option for MPM patients is available. The failure of the phase 2 placebo-controlled study of defactinib (VS-6063) in subjects with MPM (COMMAND), taken together with our finding that FAK inhibition can interfere with MPM spheroid growth in vitro and tumor growth and angiogenesis in vivo, highlights the importance of exploring combination therapeutic options to fully exploit the anti-tumor activity of FAK inhibitors in this fatal malignancy.

## Conclusion

In conclusion, by investigating a large panel of MPM cell lines, we found that BI 853520, a novel, highly selective FAK inhibitor, has only limited activity in adherent 2D cell cultures, but it effectively inhibits the growth of 3D tumor spheroids. Moreover, BI 853520 blocked the growth of orthotopically growing human MPM xenografts in vivo. Additional studies are needed to further delineate the therapeutic value of BI 853520 in MPM.

## Electronic supplementary material

Supplementary Fig. 1 **Histological subtype and Merlin status do not determine BI 853520 sensitivity of MPM cells in vitro.** Following treatment with different concentrations of BI 853520, MPM cells were incubated for 72 h and their viability was assessed by SRB assay. BI 853520 IC_50_ values were determined for 12 MPM cell lines. No correlation between BI 853520 IC_50_ histological subtype (a) or merlin status (b) was found. (PNG 246 kb)

High Resolution Image (TIF 54.8 kb)

Supplementary Fig. 2 **Effect of combination treatments with BI 853520 and cisplatin on MPM cell viability.** Following treatment with different combinations of different concentrations of cisplatin and BI 853520, SPC212 and P31 MPM cells were incubated for 72 h and their viability was assessed by SRB assay. There were no consistent synergisms observed between cisplatin and BI 853520 treatment regimens. (PNG 732 kb)

High Resolution Image (TIF 115 kb)

Supplementary Fig. 3 **Effect of FAK inhibition on intracellular signaling pathways in adherent MPM cells.** The densitometry of the time-course immunoblot assay (Fig. 3) shows that 1 μM BI 853520 treatment induced an effective and durable inhibition of the phosphorylation of FAK. In contrast Erk activation was only reduced in P31 cells and at the 24 h there was no difference to the control. Akt phosphorylation was not reduced in any of the cell lines. The inhibition of S6 phosphorylation was also not durable in any of the cell lines studied. As loading control β-tubulin was applied. (PNG 407 kb)

High Resolution Image (TIF 50 kb)

Supplementary Fig. 4 **Quantification of the effect of BI 853520 on FAK phosphorylation and downstream signaling pathways in MPM spheroids.** The left panel shows the immunoblot assays depicting the impact of 24-h 1 μM BI 853520 treatment (indicated by +) on FAK, Erk1/2, Akt and S6 phosphorylation in SPC212, SPC111, P31 and M38K spheroids. As loading control, β-tubulin was applied. The densitometry quantification indicates that FAK phosphorylation was potently inhibited in all four MPM cell lines. In contrast, the phosphorylation of Erk1/2, Akt and S6 was not reduced in these four MPM cell lines. Phosphorylation of Akt was not detectable in P31 and M38K spheroids irrespective of treatment. (PNG 805 kb)

High Resolution Image (TIF 167 kb)

Supplementary Fig. 5 **BI 853520 does not specifically target tumor-initiating cells in MPM spheroids.** MPM spheroids were treated with BI 853520 for 4 days and the mRNA expression of tumor stem cell markers were analyzed by qPCR. GAPDH was used as reference gene. Transcript levels (mean ± SD) from two independent experiments are presented.. (PNG 1076 kb)

High Resolution Image (TIF 174 kb)

Supplementary Fig. 6 **BI 853520 does not induce apoptosis in orthotopically growing human MPM tumors in mice.** (a) Apoptotic MPM cells (green) in BI 853520- and solvent-treated tumors. DAPI (blue) was used as nuclear counterstain. Scale bar: 100 μm. (b) Quantification of the TUNEL-positive MPM cells as percentages of all DAPI labeled cells demonstrates the lack of effect of BI 853520 treatment on tumor cell apoptosis. (PNG 996 kb)

High Resolution Image (TIF 183 kb) (TIF 222 kb)
