# Supplementary figures and images for: The FAK inhibitor BI 853520 inhibits spheroid formation and orthotopic tumor growth in malignant pleural mesothelioma
Source: J Mol Med (Berl). 2018 Dec 11;97(2):231–42. doi: 10.1007/s00109-018-1725-7 (PMC6348072; doi:10.1007/s00109-018-1725-7)

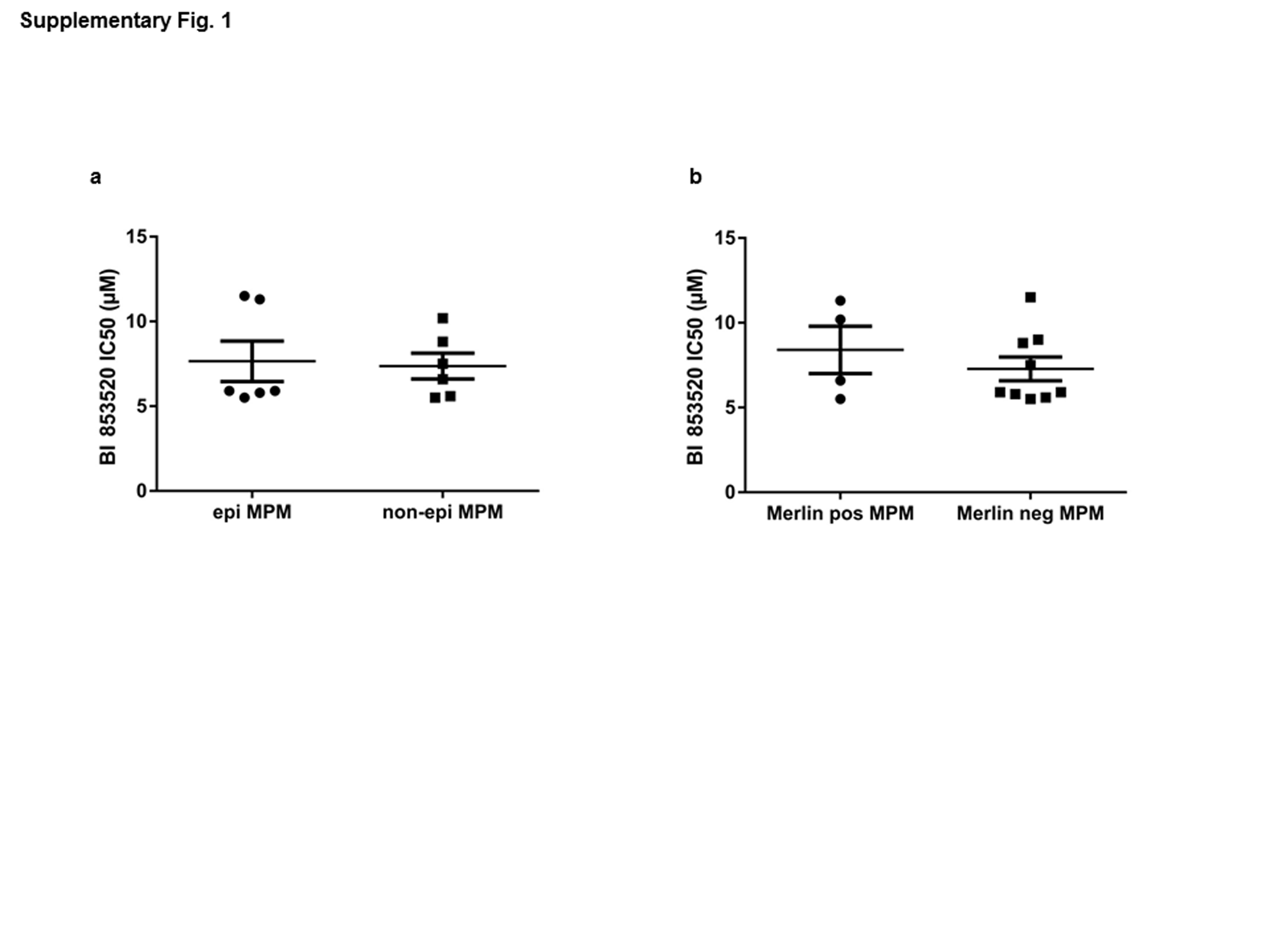

Supplement: Supplementary file 1 — Histological subtype and Merlin status do not determine BI 853520 sensitivity of MPM cells in vitro. Following treatment with different concentrations of BI 853520, MPM cells were incubated for 72 h and their viability was assessed by SRB assay. BI 853520 IC50 values were determined for 12 MPM cell lines. No correlation between BI 853520 IC50 histological subtype (a) or merlin status (b) was found. (PNG 246 kb) [file 109_2018_1725_Fig7_ESM.png]

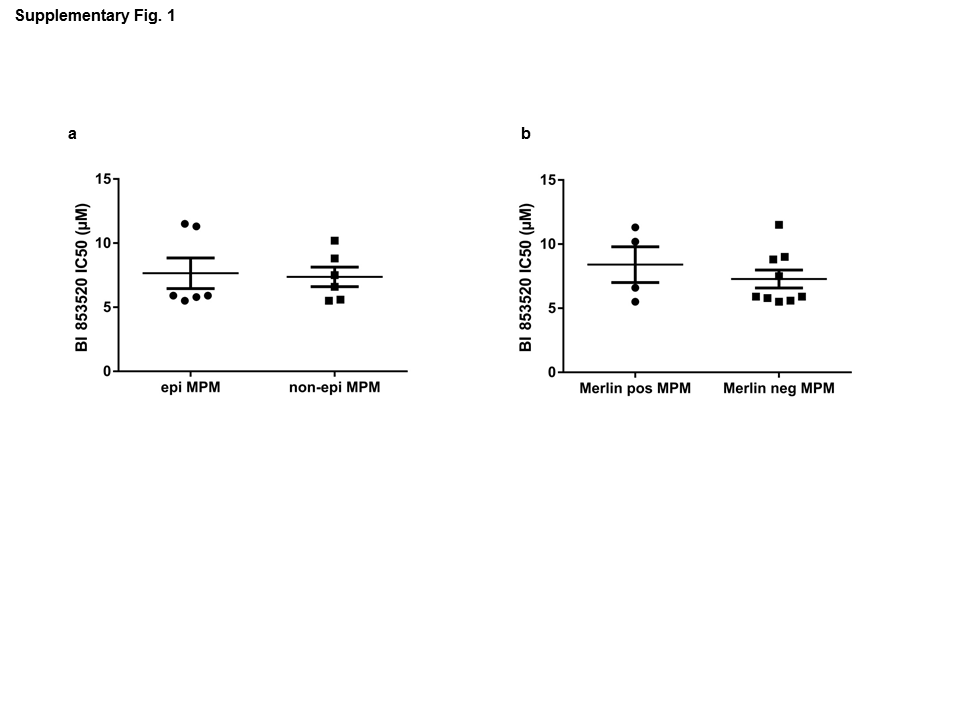

Supplement: Supplementary file 2 — High Resolution Image (TIF 54.8 kb) [file 109_2018_1725_MOESM1_ESM.tif]

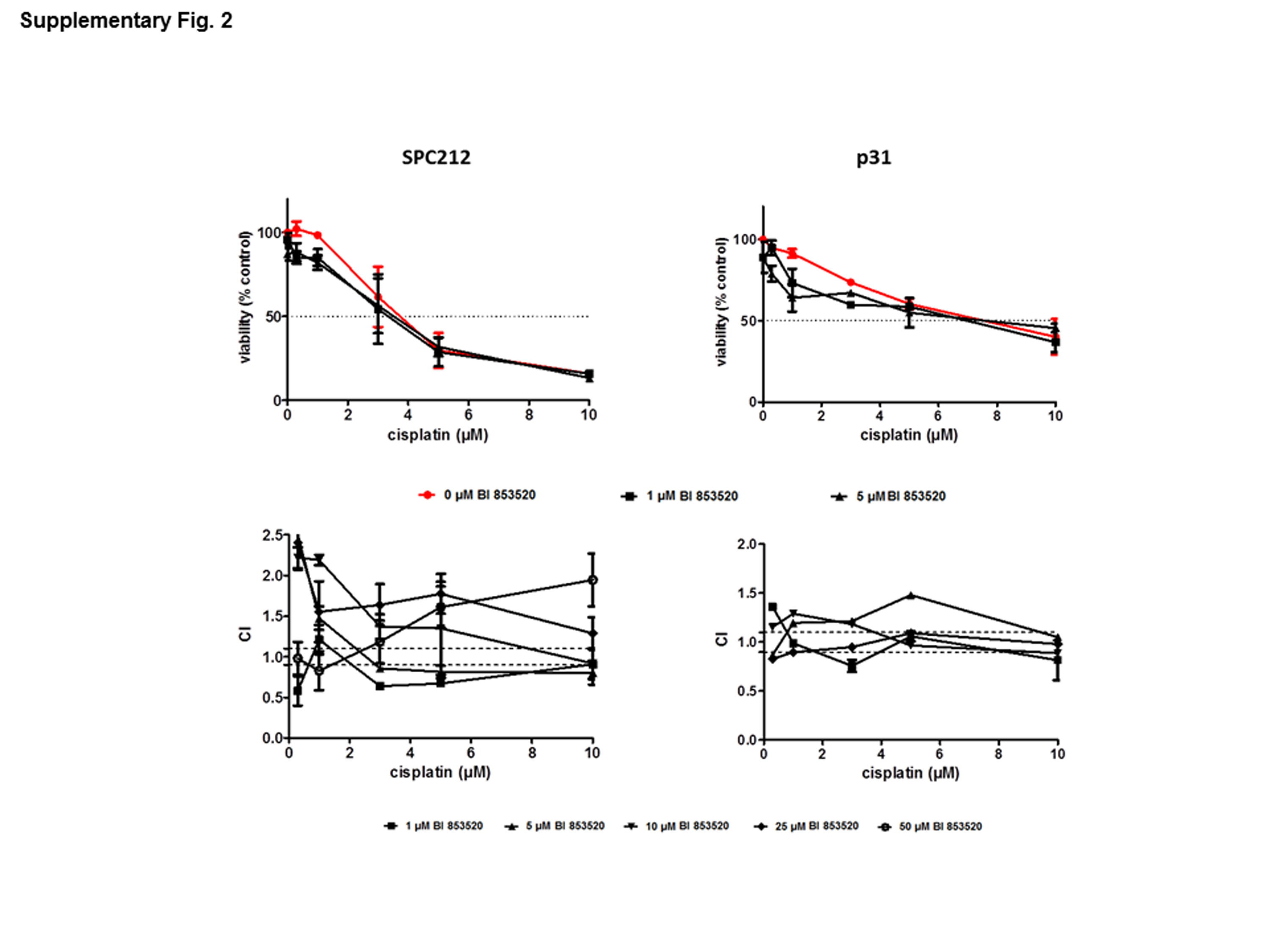

Supplement: Supplementary file 3 — Effect of combination treatments with BI 853520 and cisplatin on MPM cell viability. Following treatment with different combinations of different concentrations of cisplatin and BI 853520, SPC212 and P31 MPM cells were incubated for 72 h and their viability was assessed by SRB assay. There were no consistent synergisms observed between cisplatin and BI 853520 treatment regimens. (PNG 732 kb) [file 109_2018_1725_Fig8_ESM.png]

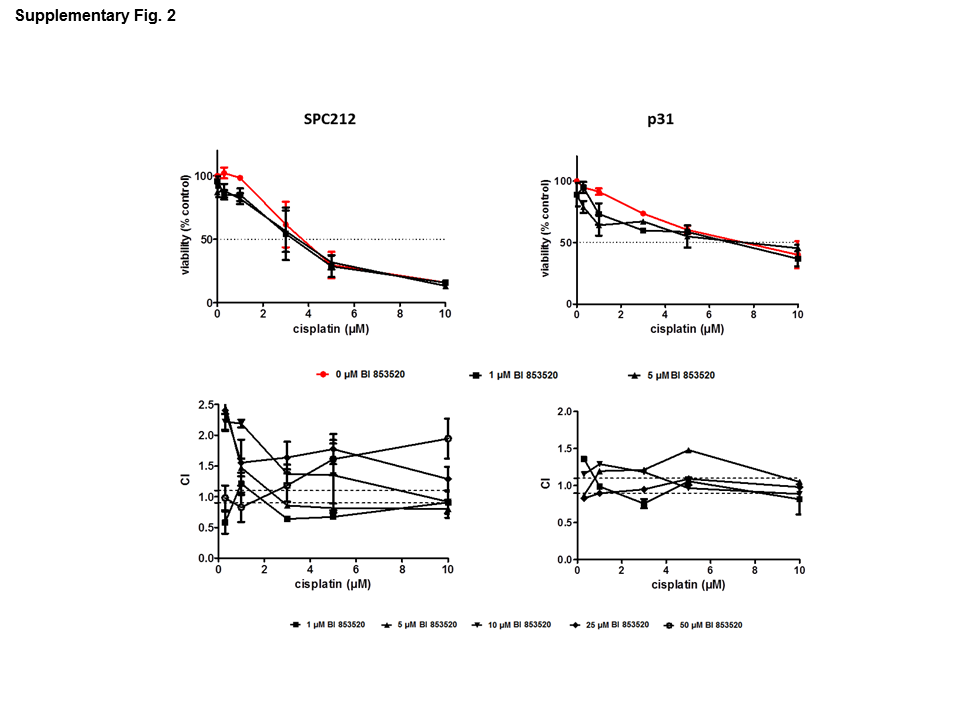

Supplement: Supplementary file 4 — High Resolution Image (TIF 115 kb) [file 109_2018_1725_MOESM2_ESM.tif]

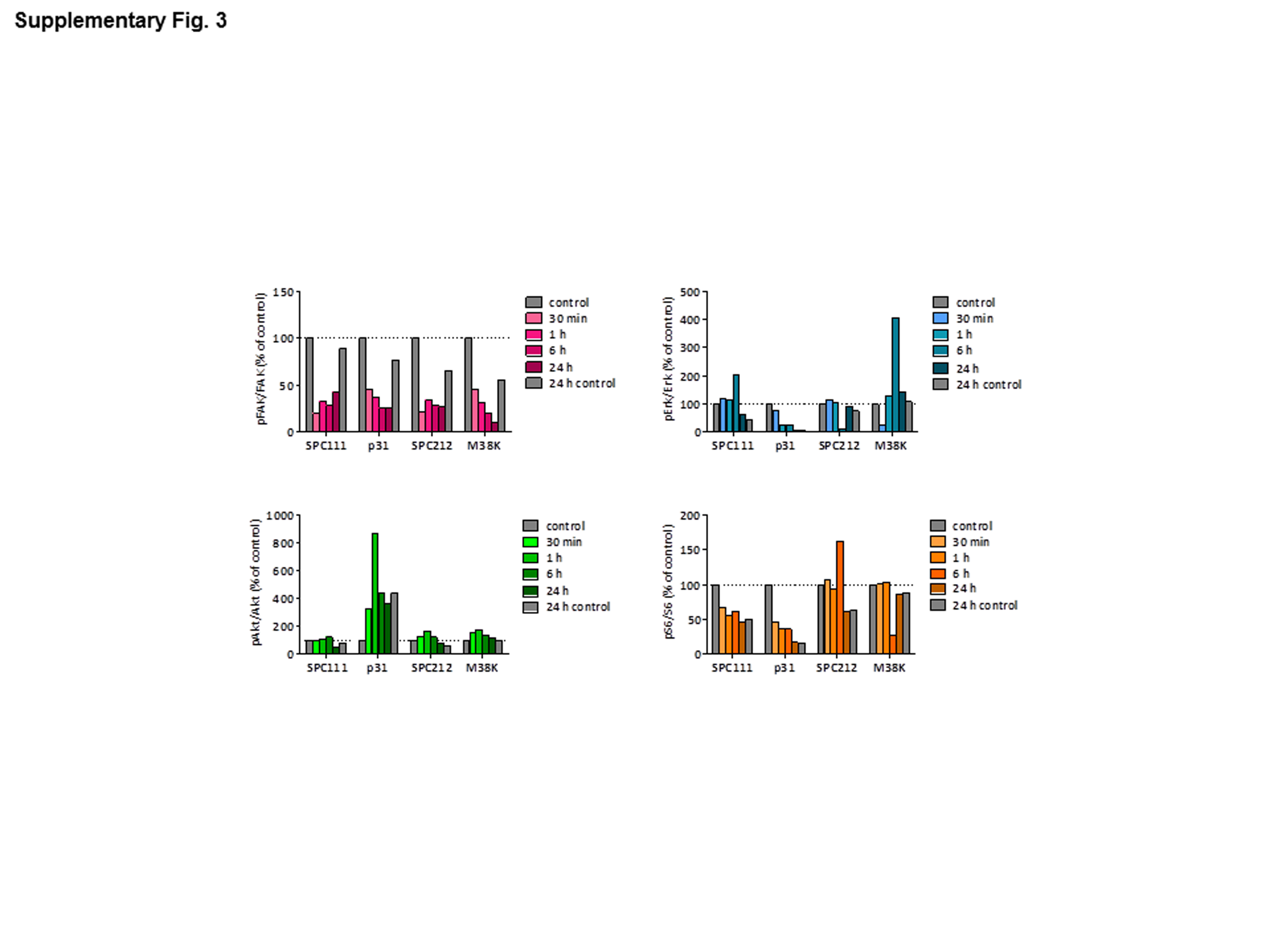

Supplement: Supplementary file 5 — Effect of FAK inhibition on intracellular signaling pathways in adherent MPM cells. The densitometry of the time-course immunoblot assay (Fig. 3) shows that 1 μM BI 853520 treatment induced an effective and durable inhibition of the phosphorylation of FAK. In contrast Erk activation was only reduced in P31 cells and at the 24 h there was no difference to the control. Akt phosphorylation was not reduced in any of the cell lines. The inhibition of S6 phosphorylation was also not durable in any of the cell lines studied. As loading control β-tubulin was applied. (PNG 407 kb) [file 109_2018_1725_Fig9_ESM.png]

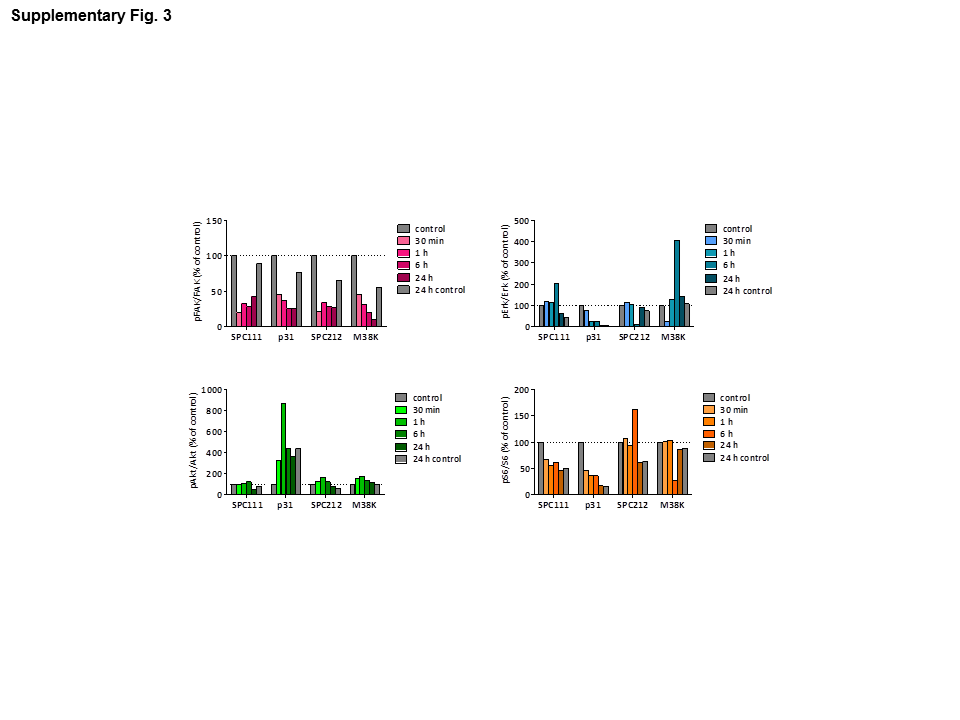

Supplement: Supplementary file 6 — High Resolution Image (TIF 50 kb) [file 109_2018_1725_MOESM3_ESM.tif]

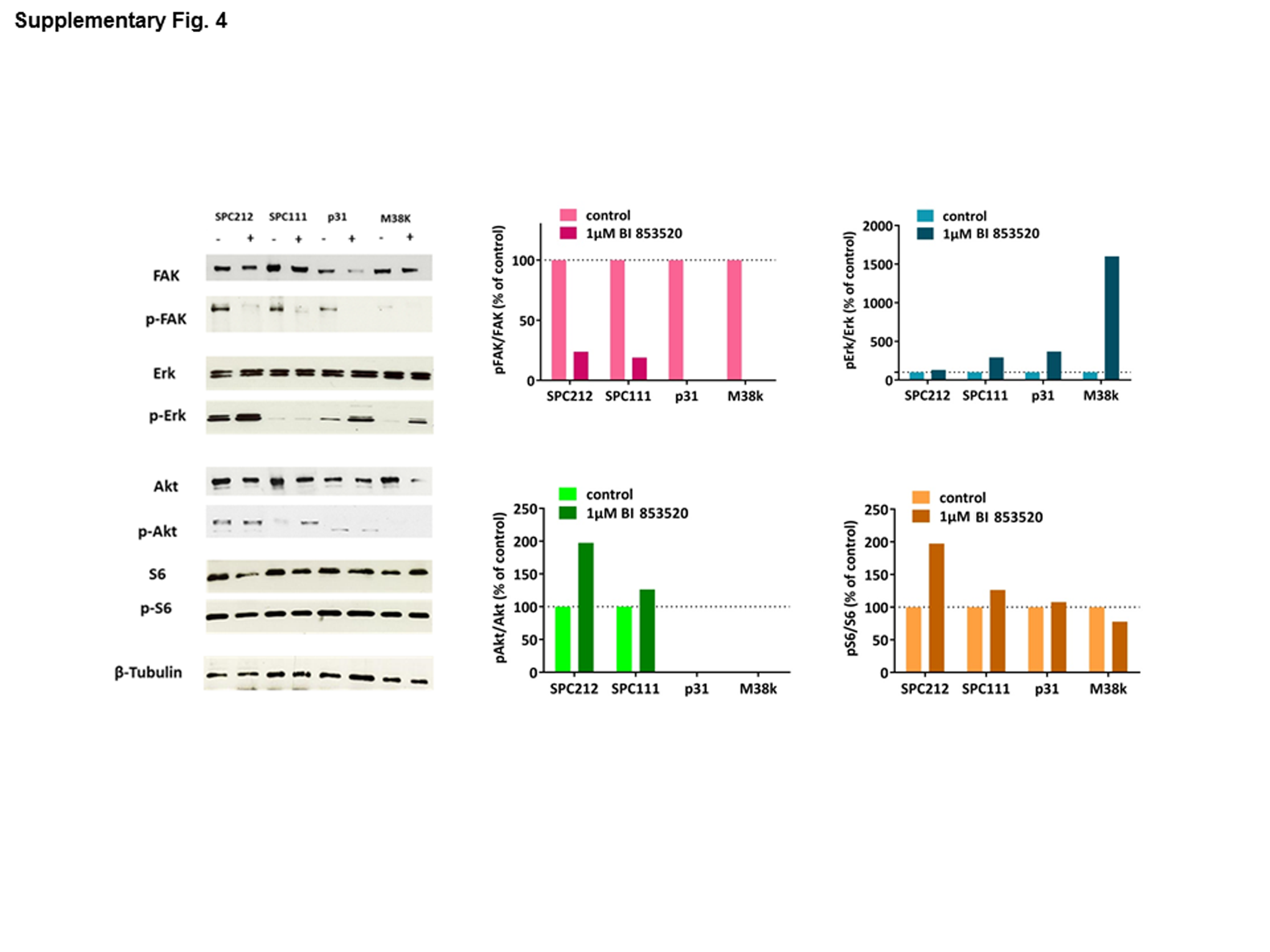

Supplement: Supplementary file 7 — Quantification of the effect of BI 853520 on FAK phosphorylation and downstream signaling pathways in MPM spheroids. The left panel shows the immunoblot assays depicting the impact of 24-h 1 μM BI 853520 treatment (indicated by +) on FAK, Erk1/2, Akt and S6 phosphorylation in SPC212, SPC111, P31 and M38K spheroids. As loading control, β-tubulin was applied. The densitometry quantification indicates that FAK phosphorylation was potently inhibited in all four MPM cell lines. In contrast, the phosphorylation of Erk1/2, Akt and S6 was not reduced in these four MPM cell lines. Phosphorylation of Akt was not detectable in P31 and M38K spheroids irrespective of treatment. (PNG 805 kb) [file 109_2018_1725_Fig10_ESM.png]

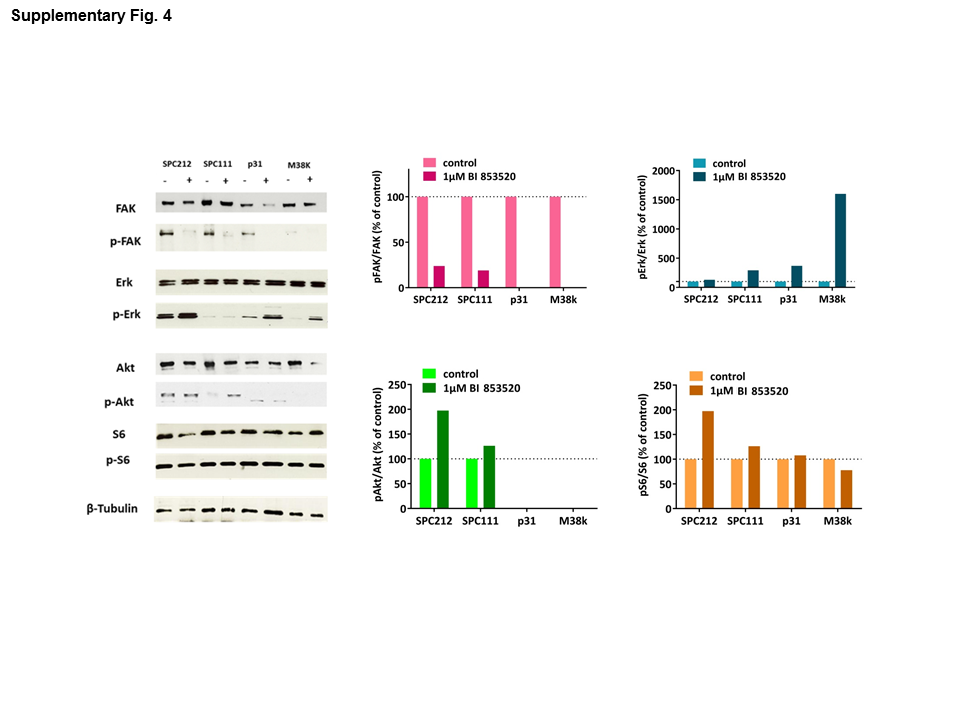

Supplement: Supplementary file 8 — High Resolution Image (TIF 167 kb) [file 109_2018_1725_MOESM4_ESM.tif]

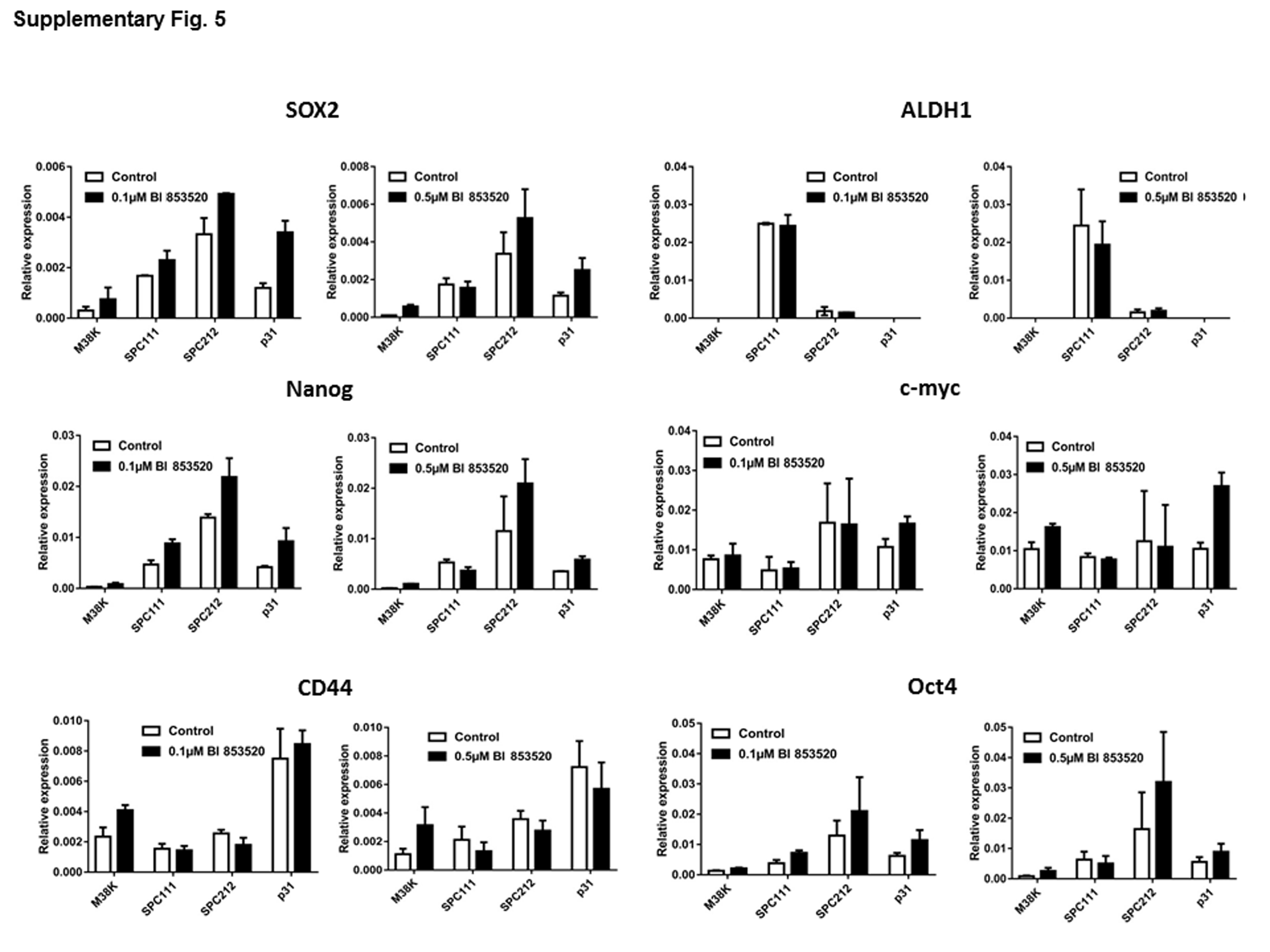

Supplement: Supplementary file 9 — BI 853520 does not specifically target tumor-initiating cells in MPM spheroids. MPM spheroids were treated with BI 853520 for 4 days and the mRNA expression of tumor stem cell markers were analyzed by qPCR. GAPDH was used as reference gene. Transcript levels (mean ± SD) from two independent experiments are presented.. (PNG 1076 kb) [file 109_2018_1725_Fig11_ESM.png]

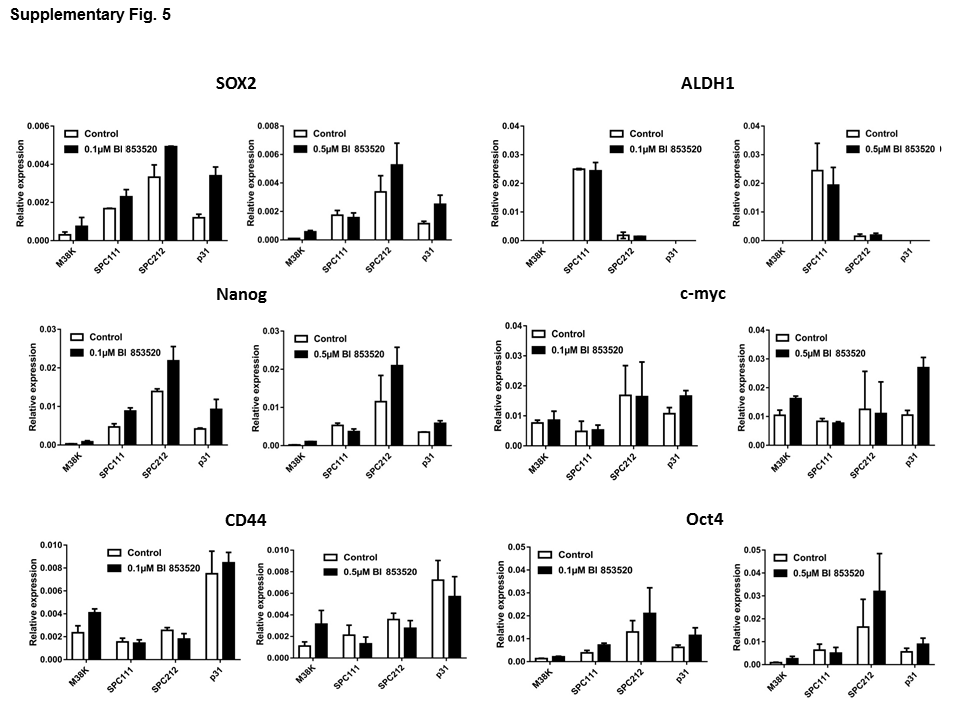

Supplement: Supplementary file 10 — High Resolution Image (TIF 174 kb) [file 109_2018_1725_MOESM5_ESM.tif]

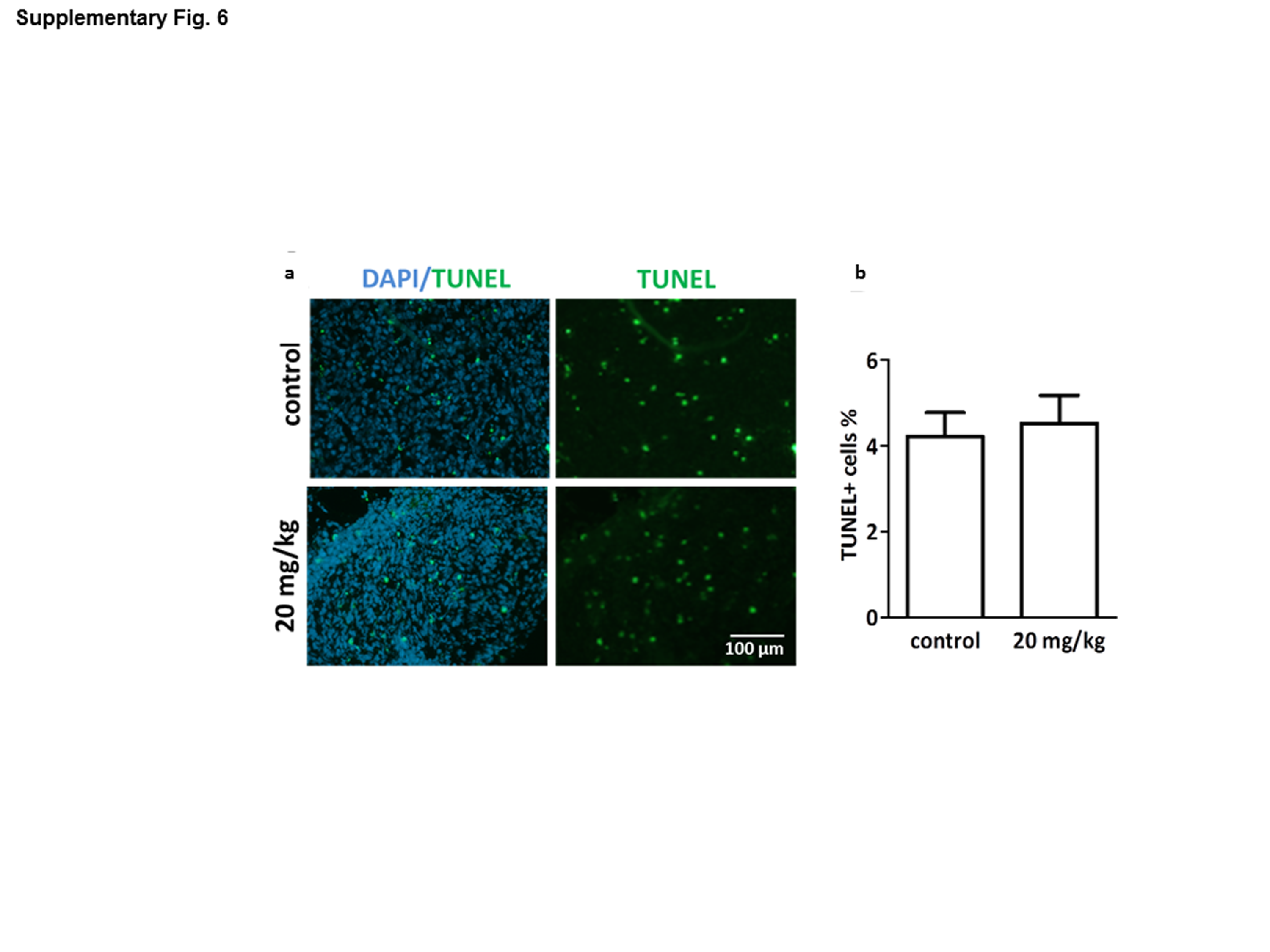

Supplement: Supplementary file 11 — BI 853520 does not induce apoptosis in orthotopically growing human MPM tumors in mice. (a) Apoptotic MPM cells (green) in BI 853520- and solvent-treated tumors. DAPI (blue) was used as nuclear counterstain. Scale bar: 100 μm. (b) Quantification of the TUNEL-positive MPM cells as percentages of all DAPI labeled cells demonstrates the lack of effect of BI 853520 treatment on tumor cell apoptosis. (PNG 996 kb) [file 109_2018_1725_Fig12_ESM.png]

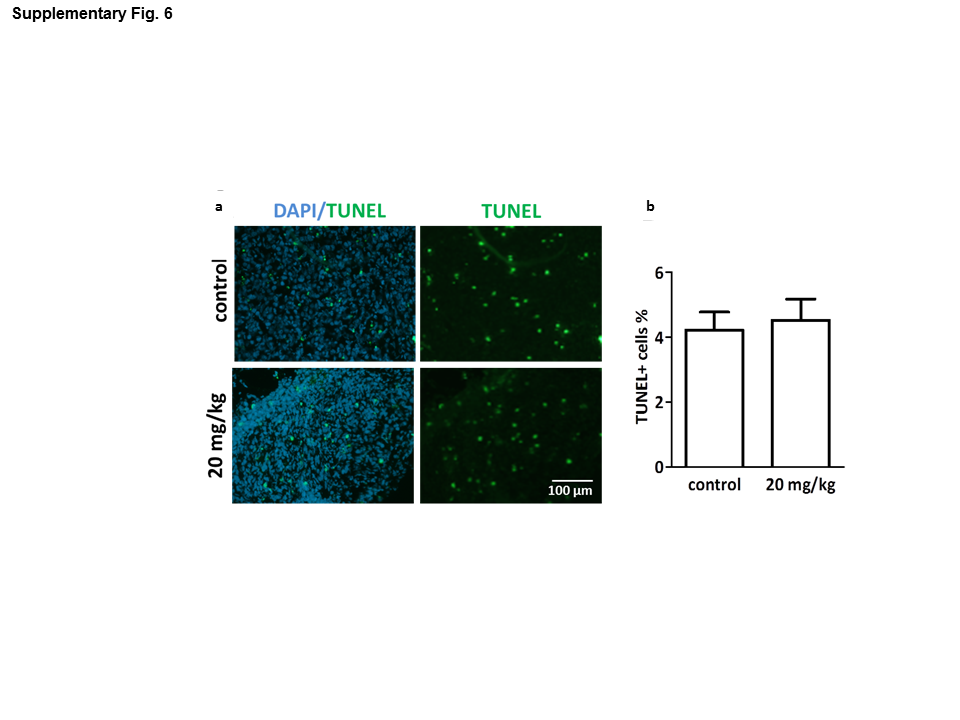

Supplement: Supplementary file 12 — High Resolution Image (TIF 183 kb) (TIF 222 kb) [file 109_2018_1725_MOESM6_ESM.tif]
